# Turning the World Upside Down: Playing as the Deliberate Creation of Uncertainty

**DOI:** 10.3390/children1020241

**Published:** 2014-09-12

**Authors:** Stuart Lester, Wendy Russell

**Affiliations:** Applied Sciences, University of Gloucestershire, Oxstalls Campus, Oxstalls Lane, Gloucester GL2 9HW, UK; E-Mail: wrussell@glos.ac.uk

**Keywords:** play, risk, childhood, health, well-being, biopolitics, posthumanism, relationality, materiality, performativity

## Abstract

Risk is big business. It has assumed almost universal acceptance as an ever-present reality of life, something out there waiting to cause harm (most notably to political, economic and health systems). It commands vast resources to develop preventative measures that are the preserve of experts issuing often contradictory advice and warnings. Children’s play is caught up in this account. No longer something that children just do, it is subject to adult scrutiny that simultaneously and paradoxically attempts to manage risk and promote “risk-taking” for its perceived instrumental benefits, primarily the development of risk assessing skills. Adults thus guide children’s play, rendering children passive and needy recipients of expertise. This article takes a broader perspective to consider how this contemporary understanding of risk plays out in material discursive practices in relation to childhood, play, health and wellbeing. It then draws on conceptual tools of relationality, materiality and performativity to reconfigure playing as an emergent co-production of entangled bodies, affects, objects, space and histories in ways that make life better for the time of playing. Such moments produce health-affirming potential as an intra-dependent phenomenon rather than an individual achievement. Finally, it considers implications for “health promotion” and health enabling environments.

## 1. Introduction

*One summer afternoon, some children had been investigating around the edges. One boy emerged with the red plastic slide from the kit house that is scattered around. He said “Look what I found! What can I do with it?” Several other children followed him. They decided to take it up the water tower structure. They worked together to lift the slide up the structure. They got to the level where the rope hangs over the sand pit. The group of 4–5 boys involved were all very competent climbers so I decided to watch from a distance what happened next. They pushed the slide out over the end of the structure above the sand and two of them sat on the slide, stopping it from falling over the edge with their weight. Then after a countdown, the boy at the back got off and the slide dropped with one boy still on it. He grabbed the rope just in time to stop himself falling along with the slide. The level of excitement was something I’ve not seen before on the playground. He climbed down. The other boys congratulated him on surviving. He said “That was sick! That was sick you know!” One of the other boys said* *“We could do this every day!” The first boy said* *“I didn’t know I was going to make it! I thought I was going to die!” (Research participant’s blog* [1]*).*

We can make sense of this extract from a blog in a recent action research project on an adventure playground in a number of ways. The aim of this article is to present a perspective on playing that challenges and extends the current common-sense understanding of “play” and its instrumental application in policy and practice settings, using this extract as an illustration. The endeavor here is to turn conventional wisdom on its head in pretty much the same fashion as children do when playing. In performing this task, the intention is not merely to critique and deconstruct, but to reconfigure, by drawing on a different set of conceptual tools and approaches from those traditionally employed in the study of play and by doing so attempt to forge some new connections. It is a generative and additive piece, assembling ideas that are intended to multiply rather than subtract [2] and to open what appears to be taken-for-granted assumptions and relationships to more critical scrutiny to see what more might be revealed. In undertaking this task, the article:

- considers contemporary perspectives on risk and how they play out in material discursive practices in relation to childhood;
- explores the entanglement of play, risk and health;
- introduces another perspective on playing; and
- considers implications for “health promotion” and health enabling environments.

## 2. Risky Childhoods

Pre-modern meanings of risk largely portray it as existing outside of human affairs; humans could do little against these potential dangers other than estimate their likelihood and take steps to limit their impact [3]. Its origins in modern usage can be attributed to principles of maritime insurance as a way of describing the balance between opportunities for profit and potential dangers [4]. This suggests a relatively neutral position on risk, implying there are potential benefits as well as losses. However with the advancement of “modernity” (the rise of an industrialized, scientific, technical, rational and liberal state), risk is commonly equated with threats and adverse outcomes. While definitions and applications may be contested, in the context of this article it is used as a generic concept that denotes “a family of ways of thinking and acting that involve calculations about probable futures in the present followed by interventions into the present in order to control that potential future” [5] (p. 70). The risks apparent in the opening scenario therefore might include serious injury on the part of the child, or allegations of negligence on the part of the adult observer (perhaps in the form of legal action), both of which require an intervention to reduce or remove this possibility. It is this interventionist and utopian discourse that is central to the discussion here. First, we propose an opening position, drawing on and extending Foucault’s [6] concepts of biopower/biopolitics and governmentality, tracing this through more contemporary thinking that engages with “risk” [3,5,7,8]. It considers how complex and multiple disciplinary forces, coalescing around notions of risk and enshrined in so many socially legitimated powers and authorities, seek to shape and fashion the lives of individuals [8,9].

Risk is a defining feature of modern society and pervades all aspects of everyday life, filling it with perceived physical, moral, psychological, social, technological, economic, geopolitical and environmental dangers [10]. Scientific advancements have unveiled numerous previously unknown risks, bringing them to our attention thereby also creating demand for action against them. Risk has become the lens through which activities are judged, yet such judgments are now largely beyond the lay-person [11]. The science of risk calculation, assessment and evaluation has become the hallmark of modernity’s progress by rationalization and calculation; “from the actuarial tables of life insurers to the risk analysis of those in the business of risk: the movers and shakers of capitalism” [12] (p.12). The technology of risk-assessment is entangled with knowledge, instruments, bodies, institutions and spaces to form assumptions about life itself [5] and to shape patterns of governance. The biopolitical drive to minimize risks to human health extends, for example, to control of environmental pollution, reduction of accidents (including falling off the plastic slide in the opening scenario), maintenance of body health and nurturing of children.

A key feature of the biopolitics of risk is the governing of conduct [9]; people are placed under constant surveillance while at the same time increasingly encouraged to monitor themselves. It marks a political and ethical field where individuals are obliged to assess, make responsible choices and to take control over their lives, to monitor inputs (food, sleep, alcohol, nicotine, *etc*.) and outputs (exercise, time-management, body shape, *etc.*) with the intention of minimizing exposure to health hazards [8]. Failure to do so labels individuals as “risky”, generating both societal disapproval and also potentially feelings of personal shame, despair or disengagement [13]. Rose calls this the “responsibilization” of life, or what Beck refers to as “individualization”, in which more and more aspects of behavior are subject to self-reflection and self-management. Thus, for example, family support networks are replaced by reliance on individual ingenuity to develop personal support mechanisms and economic self-responsibility [10]. Evaluating risk establishes a moral dimension to bodily behavior, creating a hierarchy between those who choose to use the advice on “safe” ways to manage their bodies and those who do not. Individuals are encouraged to “care for the self” and blame may be attached to those who fail or who choose not to take responsibility for their own health.

This “modern” conception of risk inevitably contributes to the formation of childhood, marking it as a period of the life-course in which the vulnerable innocence of the child needs protecting from the multiple risks that lie in wait to cause harm [14]. There is an inherent presumption that children’s vulnerability and immaturity render them more susceptible to risks than adults who are better positioned to make informed judgments [15]. Returning to the opening scenario, the observing adult is understood as being responsible for making judgments about the likelihood of serious injury, evident in her comment about the boys being competent climbers. The construction of the innocent child imbues children with their own form of “specialness”, [15]. Adult nostalgia for times spent playing outdoors, in a carefree state away from adults, provokes a sense of loss for more innocent times. Perceived contemporary social ills threaten this state of innocence and promote ever-increasing levels of risk-anxiety and fear (for example, child abuse, children’s access to information technologies and the commodification and sexualization of childhood). In minority world countries, contemporary childhood has become the most intensely governed period of life [16]. (We use the terms “minority” and “majority” here to refer to what are often termed “developed” and “developing” countries respectively. This format is preferred as it acknowledges that much power resides in the few countries whose economic, political and cultural activities affect the majority of the world. We are also aware that the use of these terms may suggest a dichotomy that elides the multiple and diverse contexts and contestations that ebb and flow between such a simple division.) Children’s lives are increasingly subject to measuring and monitoring to provide “more accurate measures of the conditions children face and the outcomes various programs achieve” [17] (p. 21), giving rise to high levels of surveillance in which the “child has become the target of social, political, educational and legal regulations that constitute children as the powerless and dependent Other in relation to adults in society” [18] (p. 5) and as such in need of protection. The discourse of protection, generally framed within the well-intentioned notion of acting in the best interests of the child (who would not want children’s lives to be better?), impacts in multiple and complex ways to shape how children are perceived and acted upon in the family, school and in wider society [18].

Not only are children’s positions fixed; adults, as the protectors of children, need to be scrutinized and made accountable, carefully regulated to avert any threat to children’s innocence [19]. The regime of risk management acts as a regulatory technology that determines what is desirable and acceptable. It is enacted through a series of judgments and comparisons (policies and standards) and associated practices of symbolic and material rewards and sanctions that come to represent the worth and value of individuals and organizations. Professional practice becomes framed in an over-riding sense of prevention:

*We used to have the kids out running around clothed only in their suntan [lotion], naked under those on a hot day. Now we wouldn’t do that. We are aware of cultural issues, cultural safety, some cultures don’t like them naked, but also sun safety, and of course the safety from* *voyeurs*.

[20] (p. 242)

Thus, the discourse of risk has material consequences and is played out and negotiated in everyday relationships and spaces; parental anxieties and responsibilities may delimit children’s ability to negotiate time/space away from adults [21,22,23]; practitioners are guarded in their contact with children [20,24].

The governance of children is not just about maintaining the discourse of childhood innocence. Childhood represents a projection of adults’ desires, hopes and fears, rendering children redemptive agents who hold the promise of becoming better and who need careful investment in order to realize the utopian vision [25]. It has become a state project of control through particular configurations of language, institutions, materials and space, or what Deleuze and Guattari [26] term “molar assemblages”. These seek to shape children’s minds and bodies in order to ward off any possible risk to this progression towards the compliant and consuming citizens of tomorrow. The responsibility for safe progression falls to the institutions of childhood (primarily family, school, nursery and health centers) which combine to form a “plane of organization” [26], or blueprint of ideal development, where technical accounts of well-being are increasingly applied to measure progress. These institutions are the conduit through which lives are governed [27].

Health and education institutions have co-emerged as central pillars of this project and have increasingly spread their regimes and accounting procedures into other sectors [28]. The foundations of biomedical accounts of health, (generally seen as the absence of disease) and development (generally seen as teleological progression) are deterministic or reductionist in establishing cause-effect relationships [8]. Universal norms are drawn from limited studies to generalize solutions for a range of biomedical, psychological and social risks and problems. The continuous refinement of accounting systems ensures that children can be measured and monitored in systematic ways.

A biomedical perspective also assumes a particular construction of “the body”, as a relatively stable thing that is pre-social and pre-discursive, ready to be over-coded by adult calculations and interventions aimed at normalizing “health consciousness”. Western philosophical underpinnings of thought, in which cognition is held to be superior to the unruly body, dominate approaches to education and health; the mind is something to be cultivated, and by making conscious, informed, “right” choices the body and its affects are to be controlled, policed, subdued and got out of the way [29]. Such an approach privileges rationality and autonomy; it becomes “an instrumental, calculating and totalizing reason and a scientific knowledge that is unified and claims to reveal an objective and universal truth about humanity, history and nature” [30] (p. 230) producing a biomedical account of the body as “both the object of risk and the subject of risk-reduction” [13] (p. 123).

Children’s play is caught up in this future-focused, bio-political, technical yet nostalgic and redemptive project, and the following section considers how it has become entangled in the material discursive practices of risk, health and well-being.

## 3. Play, Health and Well-Being

In minority world countries play is held to be a defining feature of childhood, largely valued for the contribution it makes to “healthy” development. Traditional accounts portray development as a maturation process achieved by the progression through universal stages from simple to increasingly complex, or from “immature” to “mature”. The framework of development as progress proposes scenarios in which the future is known, and thus pre-exists the unfolding of life [31]; development becomes a process of “achieving full potential” or becoming filled with what a child needs to become adult. In a desire to avoid uncertainty and risk, uncritical, accepted wisdom and conventions assume a “taken-for-granted sense that harbors given solutions that correspond to given problems and given answers that correspond to given questions” [32] (p. 82). This common-sense, or orthodoxy of materials, codes, practices and discourse, presents a certain view of childhood that informs judgments about progress, distinguishing between a series of binary relationships such as right/wrong or good/bad, carried out with good intentions and in the best interest of all.

Play can be commandeered to support this progression [33] thereby assuming an instrumental value that promotes desirable play behaviors—those that clearly contribute to growing up—while at the same time censuring apparently purposeless, trivial and other undesirable play forms. Play is held to be beneficial for developing physical, cognitive, social and emotional skills, a “deferred benefits” approach in which play serves something outside of playing [34,35]. In this account, play is defined and classified as an activity, subsumed into the plane of organization, ordered, structured, and situated in dedicated time/spaces and for specific purposes. For example, a discourse of play and learning purports to welcome children’s freedom to discover and explore through play, but such freedom is held in check by pedagogical gaze and scrutiny; children’s freedom to discover is strictly monitored and controlled as it is essential that children are discovering the right things [36].

This reified, instrumentalized progress narrative extends to two interrelated aspects of interest to the discussion in this article, namely those of health (as the absence of illness) and safety (as the management of risk). Much of the focus for this is on physical, outdoor play as a particularly promoted category. One example of the growing interest in the instrumental value of play from public health institutions and health promotion units is the promotion of play as a tool to combat obesity [37,38], now given the status of an epidemic in minority world health agendas and increasingly seen as a global issue [39]. The discourse of obesity, from the normative position established by biomedicine, emphasizes the causal relationship between inactivity, poor diet, obesity and poor health; “obese and ‘at-risk’ (*i.e.*, overweight) bodies are constructed as lazy, expensive, and in need of expert control” [40] (p. 228).

The intention is not to present a critical examination of the obesity discourse; what is at issue here are the ways in which play gets caught up in this account. As Alexander *et al.* [37] note, it becomes a serious activity that requires deliberation and planning to ensure it achieves its intended purpose. The promotional literature on children’s play and obesity constructs play as a health activity, not only seeking to delimit valuable forms of play (and by implication undesirable forms, which in this context generally means sedentary) but also holding adults to account for children’s participation in such activity [37,38]. Yet by doing so such policies largely ignore how children co-create moments of play anywhere and everywhere [41]:“where children are is where they play” [42] (p. 10). Playing with the slide on the “water tower” in the opening scenario was not a deliberately planned activity aimed at promoting participation in physical activity.

Alongside the instrumentalization of “physical play” as a tool for combatting obesity is the value attributed to “risky play” and the proposed contribution this makes to children’s development of risk-assessment competencies [43]. This position is somewhat problematic and ambiguous. Under the general rubric of the protection of innocence, children’s risk-taking is seen as threatening and children are required, as a measure of increasing competence, to avoid risk; injuries and lifestyle-related illnesses are largely attributed to poor risk-management on the part of adults [44]. At the same time, a degree of risk-taking is advocated as beneficial. The development of a risk-benefit approach seeks to adopt a balanced attitude, particularly in UK:

*those responsible for play provision can develop an approach to risk management that takes into account the benefits the provision offers to children and young people as well as the risks. It aims to help providers achieve two objectives that are fundamental in any play provision: to offer children and young people challenging, exciting, engaging play opportunities, while ensuring that they are not exposed to unacceptable risk of harm*.

[45] (p. 8)

While this is seen as countering the excessive risk-aversion seen to permeate the institutions of childhood, it is still couched in the (necessary) language of technical risk management processes, placing responsibility on adults to control what is perceived to be irrational behavior. The enactment of this is entangled in a much wider discourse already discussed of adult accountability and regulation, childhood as innocence, protection, best interests and future-citizen, with accompanying discursive effects that create the “problem” and by doing so implicitly set limits upon what can be said and done [46]. Thus, adults may often make assessments of children’s play based on a literal and risk-focused reading of its content rather than an appreciation of the symbolic, “as if” nature and vivid emotional dimensions. From a protection perspective, the possibility of any injury is undesirable, presenting the potential for harm not only to children but also to adults themselves for failing in their task of keeping children safe [34]. This thinking can readily be applied to the opening scenario. The children’s behavior can be understood as risky, in that there is a risk of serious injury; the adult has to make a judgment about the likelihood of injury, which she does by saying she knows them to be competent climbers. The children could be understood to be developing risk assessment skills, as they undoubtedly are; however, what the observation also describes is the affective vitality of the experience for the children (and intimations of this for the adult), and this aspect is picked up later.

There is growing interest in play from public health and education institutions, and the concept of “well-being” has become an adjunct to issues of health and health promotion. Accompanying this is an assumed relationship between children’s health and development, actualized in such terms as “healthy development” as shorthand for normative measurements of children’s well-being. However, concepts are poorly defined: various terms such as well-being, positive health, quality of life, and happiness are often conflated, vague, lacking definition, and used inconsistently in the literature [47,48], and have become widely critiqued [49,50]. Well-being is a highly political concept that seeks to adopt an objective, normalizing account of what it is to be “well”. When it comes to accounting for children’s well-being, measurements adopt a deficit approach: children’s well-being is measured by a “lack of” education, physical and mental health. Such a stance reinforces the needs agenda in which the identity and trajectory of children is pre-ordained and applied to determine what may be missing from being “normal”, constructing an emphasis on children as “well-becomings” rather than well-beings [51]. Morrow and Mayall [50] (p. 227) conclude that the focus on well-being “is ultimately an individualistic, subjective approach that risks depoliticizing children’s lives”; studies isolate children from their everyday worlds and experiences. Measures of “well-being” say more about the priorities and ideology of political parties than lived experiences and general definitions of well-being and happiness elide the messy, complex and contingent context of people’s everyday lives.

Children’s play is increasingly implicated in this process through a range of strategic promotions designed to inculcate values about appropriate behaviors to support progress towards economically productive and healthy adults. The issue at stake here is not so much the value of these interventions but more about the ways in which they produce a certain understanding of the relationship between children and play:

*…by regulating children’s play to be healthy and active, and thus normalising the ways in which children are encouraged to play, other relative qualities of play may be neglected. Indeed, while playing simply for fun (that is, frivolous pleasure) is considered a common experience of childhood, it appears to be less important than the more productive and explicitly active play for health*.

[37] (p. 14)

Returning to the opening scenario, such an account would foreground the benefits of the physical activity and the development of risk assessment skills but pay less attention to the significance of the final few sentences:

*He said “That was sick! That was sick you know! ” One of the other boys said “We could do this every day! ” The first boy said “I didn't know I was going to make it! I thought I was going to die!”*.

However, of course, even in such situations, while children might be regulated there are always opportunities for moments of spontaneous, unpredictable, and pleasurable acts of co-creation that enable children, and sometimes adults, to escape from the demands that others seek to impose on their behaviors and movements. In addition, it is to this perspective on playing that we now turn.

## 4. Playing Differently

So far, we have looked at the ways in which play is a matter of increasing concern in the biopolitical project of childhood, assuming significance as a form of organization of bodies. In doing so, it reduces complex and lively behavior to narrow instrumental purpose, based on a biomedical model that reflects and perpetuates a series of binary relationships: adult/child, active/sedentary, work/play, safe/risky, purposeful/frivolous, health/illness, rational/irrational, *etc.*; the first of each of these being the ideal conditions for the development of healthy identities, their binary opposites sites of concern. Life begins with a pre-established endowment (genetic predispositions) and moves, through a process of cultural acquisition, towards “terminal closure, a gradual filling up of capacities and shutting down of possibilities” [31] (p. 4). The creation, identification, measurement and classification of needs is a driving force of neoliberalism (control, commodification, consumption), reducing behavior to measurements (children need 60 minutes of physical activity each day) and feeding off these for its own purpose. It produces a metanarrative that positions childhood as risky and needy, a condition defined by multiple and omnipresent threats that are analyzed with little regard for the multiple and complex ways in which children feel about, negotiate and act upon them in their everyday lives [52]. Life itself is rarely given attention; theorists have treated it as merely consequential, the derivative representation of patterns, codes, structures or systems variously defined as genetic/cultural or natural/social, and thereby expunging life from their accounts [31]. The force and vitality of life, its exuberance and suppleness (“I didn't know I was going to make it! I thought I was going to die!”) becomes over-coded by the reactive demands and discursive/material effects of such representations [27]. Attention is given to assessing, valuing and normalizing the properties of bodies rather than seeing what they can do (“I decided to watch from a distance what happened next”).

A number of studies of risk and health [40,44,52] and broader studies of childhood [53,54,55,56] counter dominant biomedical accounts, noting little attention is given to the everyday ways in which children go on with their lives. The intention here is to develop this in order to think playing differently, to turn the world upside down, by drawing on a different set of conceptual tools that may be arranged under interwoven themes of relationality, materiality, and performativity to “explore ways of engaging affirmatively with the present, accounting for some of its features in a manner that is empirically grounded without being reductive and remains critical while avoiding negativity” [57] (p. 5). It assembles a range of diverse concepts including, but certainly not limited to, strands of materialist philosophy [26,57,58], [post] human and children’s geographies [55,59,60,], hybrid studies of childhood [53,56], life and physical sciences [8,61,62,63], critical early years and education [27,32,64], and anthropology [31]. This complex entanglement offers new ways of accounting for the world, “to take a leap forward into the complexities and paradoxes of our times” [57] (p. 54). At its core is a move away from anthropomorphism and humanism, that is, the idea that humans have a privileged place in and are set apart from the world and each other. Rather it adopts a position that presents life as emergent, multi-layered, non-linear and in a state of constant constitutive and interactive flux; “individuals do not pre-exist their interactions, rather individuals emerge through and as part of their entangled intra-relating” [61] (p. ix). This perspective on emergence offers a different viewpoint on development where time, space, bodies, materials and meanings come into co-existence and are “iteratively reconfigured through each intra-action, thereby making it impossible to differentiate in any absolute sense between creation and renewal, beginning and returning, continuity and discontinuity, here and there, past and future” (*ibid*.). It is through this process that life takes shape; there is no fixed self-contained identity but it is always creatively and actively assembled. The concern then is not about function and structure, cause and effect, but about the process of desire, that is the productive and creative force of life itself to exist and become something more [8], to realize what else bodies can do. To ask what a body can do is to pursue a line of enquiry into what particular intra-actions human and non-human bodies, materials and so on can compose [63] to produce the best possible state that conditions allow. Following Deleuze’s reading of Spinoza [65], desire marks the ability of a body to affect and be affected; affect in this sense is an expression of a body’s power to act:

*[it is] more than a feeling or an emotion it is also a potential for action, a dispositional orientation to the world. In each sense, affects are an inevitable by-product of encounters in that every encounter subtly transforms a body’s affective capacities*.

[63] (p. 627)

This brief introduction sets the foundations for thinking differently about playing and at this stage a brief observation of two children playing is introduced [66] (p. 22):

*Two young children, a boy and a girl, are sitting playing with some “gooey ” like stuff, when the following conversation occurred:*

Boy: What about if everything was made out of gooey?

Girl: Well, hmm, we would actually have all goo on our bums and stuff and we’d be all gooey and pooey and booey

*The boy laughs*

Boy: What if everything was made out of poo eugh!

Girl: Err, we would all have poo on our bums

Boy: And what about poo people?

Girl: Yuck

Boy: And what about poo willies!

Girl: No [boys name], no

Boy: What about poo trees

Girl: Yuck

Boy: What about, this is the worsest thing, what about poo leaves!

Girl: Why would you want to make poo leaves?

Boy: What if everything was made out of poo?

Girl: I dunno.

A relational and performative perspective suggests bodies and materials are continually and inextricably responsive to local conditions. It decenters the human individual as the locus of agency while acknowledging the power of things, as vibrant materials, to affect other bodies, enhancing or weakening their collective power to act [58]. These moments are singular events; a world made from poo will not occur in this manner again; it cannot be a signifier of anything other than itself as it only relates to itself as a novel formation. However the micro-details from this observation matter as they open up the possibility to look closer at this event and pose further questions and digressions [67]. The focus is on process and not codification or positionality that cuts the co-creation apart, reducing it to individual components and imposing fragmentary analysis. In this sense, playing is a phenomenon with a certain style and force (pleasurable, “as if”, indeterminate, emergent), although the very description of the event as play potentially isolates it from the flow and movement of life. A world made from poo is not a separate text [33] but is inherently situated in the environments and interwoven with and created from the materials that children encounter moment-by-moment in their everyday lives.

This restores playing as a process over the identification of a distinct and final form. It allows for more fluid, discontinuous, contingent and multiple forms of expression that pervade and persist across life [68]. No longer an exercise in accuracy or attribution of some utilitarian or instrumental developmental purpose that occurs outside of playing, attention switches to the performance of the moment. Playing has no original identity, but is emergent, and gaze is brought to bear on bodies and things co-joined in situated action [68]. Even in an apparently mundane game such as rock, paper, scissors, which appears to be a simple matter of making random arbitrary choices between three symbols, there is much more going on [69]. For example, minds/bodies may become attuned to each other to try and predict actions, and as players build experience, they may start to discern patterns. It is performative guessing and second-guessing (and third, fourth) through attending to movements, patterns, affects:

*Where is the uncertainty in Rock/Paper/Scissors? That should be obvious. It is in the unpredictability of opposing players. In fact, that is all there is in Rock/Paper/Scissors* *Rock/Paper/Scissors is a game of player unpredictability in its purest form, for this single factor is the sole determinant of the game’s uncertainty, its raison d’être, and its cultural continuance*.

[69] (p. 32)

Playing is a precarious achievement in which the material and social are entangled in all kinds of “promiscuous combinations” [70] (p. 4). It also denotes an anticipatory readiness to the environment [71], alert to the possibilities that any moment may contain for being and becoming different. The concept of becoming, in this sense, differs from the fixed trajectory of developmental psychology that children must successfully travel along to achieve maturity. Rather, following Deleuze and Guattari [26] the concept of becoming denotes a dynamic and continuous transforming relationship with the world; becoming is always a temporary combination or assemblage of heterogeneous parts each with their own intensive force that enables the emergence of new formations and affects. Attention is drawn to the ways in which mundane materials (gooey stuff), bodies (human and non-human), affects, and actions compose a moment of “what if”, a questioning of all of these elements to see what more might be done with them. Life is always in process, relational and open-ended; attention is given to pre-conscious, embodied movements and affective intensities that occur anywhere and everywhere [35,72].

This is far removed from a contemporary and increasingly pervasive disenchanted version of “what if” currently evident in approaches to risk and security, and which track through childhood, that assumes a precautionary and pre-emptive anticipatory logic [73,74]. Here “what if” questions are designed to create current solutions to perceived future social risks (the rhetoric of early intervention). However, this is problematic in ethico-political terms; projective risk mediations are based on worse case, dystopic futures and have real consequences, not simply on the individuals involved, but also “because the application of pre-emptive rationality is driving a culture in which risk scaling of people, places, and products and legal states of exception are being normalized” [74] (p. 58). Making worlds from poo may be fraught with potential dangers that may engender adult concerns: disgusting, age inappropriate, sexualized, unhygienic, and purposeless behaviors, a reductive and rational reading of irrational, irreducible and indeterminate behavior.

Yet what this fails to realize is moments of playing are affirmative and productive desires, different connections, actualizing the unexpected and by doing so temporarily breaking away from the plane of organization to become different. Bodies have a desire or incentive to be restless, moving towards the things that will increase well-being and avoiding those that decrease this state. Pursuing this, Massumi [67] notes that bodies may be distinguished by two complementary forces: they move and they feel. Thus, a body:

*…moves as it feels and it feels itself moving. Can we think of a body without this: an intrinsic connection between movement and sensation whereby each immediately summons the other*.

[67] (p. 1)

From this, the slightest movement of a body instigates a qualitative difference: movement evokes feelings and sensations that fold into each other, resonate, interfere, intensify in unquantifiable (non-representational) ways to unfold again in movement “felt and unforeseen” (*ibid*.). It marks an “accretion of feelings, capacities, opportunities and interactions” [75] (p. 149) in a particular and singular moment/event, a continuous process of dynamic change. All this can be seen in the opening scenario of the slide on the water tower structure. The following edited account from field notes from a recent action research project with an adventure playground [1] also reveals this performative process:

*Two boys (aged about 10/11) were playing a game of tag, using the circular platforms that surrounded a rope swing. It was evident that these two were part of a larger group of players, the rest safely ensconced in the hut at the top of a tower. It was also apparent that the game had a rule of not going on the ground, which constrained the two adversaries to the platform and other structures. There was also another implicit rule which meant that these two could engage in reciprocal bouts of tagging. This led to the two children standing facing each other, in very close proximity, but not touching. The person who was* *“it” would tag, and immediately receive a tag back from the other, often increasing the force of the contact in an attempt to push each other away and create a moment to flee; and then there were brief moments when both stood poised ready to tag without actually doing anything. There was a restless dance between the two, as one looked to retreat the other followed; it was almost balletic in the choreography of action, bodies and affects, tensions and laughter and so on. But this was also situated; the platforms were an integral part of this dance, and there was only one way out from the circular platform* i.e., *the walkway that led to the tower, and so the space had strategic meaning within the context of this play. Both children sought to maneuver the game to the part of the circular platform closest to the* *“escape” route and then one child decided he was going to make a break, tagged the other child and turned to run away but was pushed/tagged in return, diverting the child beyond the escape, and the other child seized the moment to run along the platform and up into the next level.*

This brief moment illustrates the sensational/motivational behaviors found in playful encounters “the ongoing, underlying process of off-balancing, loosening, bending, twisting, reconfiguring” [76] (p. 42) between bodies, things and their affects. Bodies emerge as an assemblage, connected in extensive ways and composed in recursive encounters [63], or “milieus”, composed of discontinuous movements without a beginning or end but “always a middle from which it grows and overspills” [26] (p. 23). As Duff comments [76], such milieus are important sources of developmental capabilities; intra-actions constitute affective and relational repertoires of response-abilities. They appear as ordinary events, but as Lester and Russell [34], drawing on Masten’s study of resilience [77], note, they contain properties which augment the power to act and by doing so maintain and strengthen the capacity to co-create more playful moments in the near future.

There is a growing body of research that suggests playing may contribute to the enhancement of adaptive systems, mind and body capabilities that enable life to thrive, building the capacity to cope better with uncertainty through refining stress response pathways and building a network of strong attachments to other bodies, spaces and materials. The fun and pleasure of playing generates positive affect, which has considerable health benefits and the ability to affect and be affected in a joyous manner leads to ever widening connections and greater possibilities for further connections across multiple levels of organization. Playing has been described as the deliberate creation of uncertainty [78] and as a state of “being in control of being out of control” [79] (p. 216), something that can be seen in the opening scenario, the world of poo and the balletic performance on the swing platforms. A focus on uncertainty offers a different perspective on “risky play” [80]. This generation of moderate and desirable stress which is under the control of the players may serve to prime stress response systems so there is something to draw on when faced with non-playful stress, referred to as “stress inoculation” [34].

These potential playful developmental capabilities can be understood as much more than developing skills of risk assessment, or developing resilience as an individual achievement. They become reduced when prized apart and utilized in a highly instrumental manner largely focused on the psychological and biomedical profile of resilient children and associated practices of promoting resilient capabilities. The focus on resilience as an individual capacity in an objective and measurable world relegates it to health/identity politics and the development of self-regulation [81]. The perspective on playing introduced here presents resilient capabilities in terms of desire, as a force that flows between bodies, materials and their affects “experienced in those moments of connection with life that defy common-sense, resist dominant cultural interests and power relations and in an untimely manner unsettle the identity of individuals” [81] (p. 40). MacKinnon and Derickson’s critique of the contemporary discursive effects of resilience through state agencies and expert knowledge [82] offers an alternative viewpoint that highlights its ecological, contingent and dynamic nature and brings a socio-political dimension to the discussion:

*Put another way, if alternative social relations are to be realized democratically and sustainably, and in ways that are wide-reaching and inclusive (as opposed to uneven or vanguard driven), then uneven access to material resources and the levers of social change must be redressed. To that end, we offer resourcefulness as an alternative concept to animate politics and activism that seek to transform social relations in more progressive, anti-capitalist and socially just ways. In contrast to resilience, resourcefulness as an animating concept specifically seeks to both problematize and redress issues of recognition and redistribution*.

[82] (p. 255)

The everyday environments that children share with adults are produced, regulated and over-coded with “a vast array of practices, habits, technologies, symbols and so on that constitute the maintenance routines that keep them operational” [83] (p. 45). At the same time, these generally taken-for-granted spatial orderings have exclusionary effects. As outlined in the opening section of this article, the dominant constructions and productions of risky childhood have significant influence in shaping, in contingent, complex and entangled ways, the conditions which children encounter in their everyday lives. The concept of resourcefulness switches attention from the needy and deficient child to the forces that underlie the inequitable distribution of resources and subject these to critical scrutiny, a political-ethical consideration that will be addressed in more detail in the following section.

## 5. Political-Ethical Imaginations and Health-Enabling Environments

While the forces that assemble worlds made from poo are constituted from everyday materials they are thoroughly entangled with macro forces that shape spaces and spatial practices. Moments of playfulness are not set apart from these; they work with the “real” by reconfiguring, subverting and inverting the world as it is generally given by adults to “intensify the vital productivity of daily life” [84] (p. 243). This requires consideration of adult response-ability to pay attention to equitable distribution of resources that might create the conditions in which playfulness can thrive: in other words, it is a political-ethical endeavor.

Deleuze [65] reads Spinoza’s ethics as the accumulated repertoire present at any given moment by which individuals organize their encounters to produce and maximize the experience of joyful affects, the power to live one’s life actively [85]. Ethical instances are not located or confined within the self-regulating subject but are always situated in a set of interrelations with both human and non-human materials. Ethics, in this reading, implies the ability to “cultivate, establish and sustain empowering relations as well as the commitment to the production of social conditions that are conducive to transform the negative instance into affirmative and productive ethical relations” [86] (pp. 174–175). Health, therefore, is a relational process rather than a fixed state, constituted from specific moments of connection and association of bodies, affects and materials [75]. The following observation [87] (p. 24) provides an illustration of an “ethical maneuver” carried out in a museum:

A Visitor Services Assistant (VSA) approaches a small group of children and presents them with a precious and fragile dinosaur egg (a blown goose egg) and asks them if they would take it to the VSA on another gallery. The children smile as one of them takes the egg and they carefully climb the stairs, whispering and giggling amongst themselves. At some point they find the VSA and hand over the egg. A short while later, this VSA passes the egg to another child and it starts over again.

The intention within this simple promotion is to see what more bodies and materials might do. It is a micro-political experiment from the VSA that animates the possibility of temporary escape from the molar assemblage; children are no longer passive observers of cultural artifacts, the gallery assistant no longer a supervisor of children’s behavior, the egg becomes a rare “thing” that demands care. It is an intra-active entanglement, a shared desire to simply be and become well by increasing affective capacities to act differently, a singular episode of enchantment when disciplinary power, rationality and scientific calculations are set to one side and the world is a “lively flow of molecular events, where matter is animate without necessarily being animated by divine will or intent” [71] (p. 14).

In 2013 the UN Committee on the Rights of the Child issued General Comment 17 in recognition that children’s right to play, expressed in Article 31 of the Convention, is poorly recognized by States, resulting in “lack of investment in appropriate provision, weak or non-existent protective legislation and invisibility of children in national and local level planning”. The General Comment also highlights the importance of creating time and space for spontaneous play and the promotion of societal attitudes that support and encourage playing. The Comment offers a valuable range of justifications, considerations and recommendations for improving the conditions to support children’s play [88]. However, it also implicitly contains an ethical responsibility. Responsibility in this sense means paying closer attention to the everyday movements between bodies and things and being responsive to the possibilities they contain that might help life to flourish [61]. It is a question of what can be done here and now to affect something or someone in a different way [64] to create possible futures by mobilizing resources and materials that have hitherto been overlooked or used to privilege the needs of the few over the multitude [57]. Playing reminds adults that the desire to affect and be affected “exceeds attempts to make it into an object-target for forms of power” [73] (p. 34). It presents a different version of “hope”, no longer a form of discipline and control to ensure a safe, utopian and distant future but rooted in the ordinary micro-practices of everyday life.

Such an ethical perspective challenges current biomedical accounts of health, and the ways they are played out in health promotions that seek to encourage self-regulation, a decontextualized individualization of life. The position presented here is not indifferent to the human condition, quite the opposite: “it rather implies a new way of combining ethical values with the well-being of an enlarged sense of community which includes one’s territorial or environmental interconnections” [57] (p. 10).The ideas here suggest that rather than targeting individuals, attention is given to the conditions that constitute “health enabling spaces” [75], that is, the relational, affective and material processes that intra-actively and indeterminately produce moments of care and reciprocity. The museum gallery can, through a minor experiment and for a short period of time, become a place where bodies and materials actualize different ways of being and moving beyond that of “visitor”, promoting different affects and encounters. It also opens up the possibility that there may be more of these moments to come, to actively seek out other ways of affecting and being affected.

There are no blueprints for this; ethical practices are relational, emergent and specific, but without these practices well-meaning policy/promotional prescriptions become blunt instruments. While there may be no *a priori* foundations, attention needs to be given to exploring the ways in which affects, relations, things and encounters constitute such processes; “it requires an empiricism not of identities, structures and essences but of events, processes and relations” [75] (p. 155). While the limitations of this article prohibit a detailed examination (see [83]), it is worth highlighting here the significant step taken by the Welsh Government in placing children’s play as a central component of social policy and the statutory duty for local authorities to assess and, as far as is reasonably practicable, secure a sufficiency of play opportunities. The vagueness of the term “sufficiency” defies dominant outcomes-driven policy formulation and associated technical measuring devices and calculations, providing a degree of indeterminacy that allows for the possibility of creative and experimental approaches in order to appreciate the multiple and complex processes that constitute moments of play, to build collective wisdom in order to act more responsibly with these [83].

## 6. Conclusions

This paper critiques the well-intentioned but fragmented interpretation and instrumental application of playing in biomedical and developmental psychology accounts of health and well-being and presents a counter position where playing becomes a collective self-protecting mechanism that thrives when children can create momentary time/space within their daily lives [89]. Playing (including what is sometimes called “solitary” play) is an intra-active milieu, co-creating moments in which, for the time of playing, life is simply more vibrant and there is greater satisfaction in being alive [90]. This presents a more affirmative and potentially valuable perspective in which play is not a specialized activity but rather may be seen as a creative force or desire of life itself:

*Play is the condition for the possibility of new possibility itself. To be human is to inhabit a dynamic world of not only what is but what could be Play is tension (used in this sense as a stretching out—authors note) turned toward new possibility* *without play there would be no world of meaning at all*.

[91] (p. 53)

We have a long standing and cherished recognition of individual rights, freedoms and responsibilities. Yet such a position may be untenable in the face of research from physical and life sciences that suggests all matter is lively and contains unlimited potential [58,61]. We are entwined and entangled in a complex world; there is no escape to an individual self to be viewed above or outside of this world. Our politics are constructed from the same vulnerability as the rest of life, and “to refuse to experiment is to resign oneself to the intolerable, to abandon both the struggle to change the world and the opportunity to celebrate living within it” [92] (p. 529). The issue becomes, then, one of asking what more can be done, from this perspective, to create the conditions for such affirmative “what if?” moments of health-enabling playfulness where adults can watch in wonder (and perhaps also anxiety) as children “walk the plank” on the plastic slide high up on the water tower, or co-create their balletic performances in a game of chase on the American swing, or discuss disgusting worlds of poo, or where a Visitor Services Attendant in a museum can enchant the space with a dinosaur egg.
